# Development and Application of a Polymerase Spiral Reaction (PSR)-Based Isothermal Assay for Rapid Detection of Yak (*Bos grunniens*) Meat

**DOI:** 10.3390/foods15010115

**Published:** 2025-12-31

**Authors:** Moon Moon Mech, Hanumant Singh Rathore, Arockiasamy Arun Prince Milton, Nagappa Karabasanavar, Sapunii Stephen Hanah, Kandhan Srinivas, Sabia Khan, Zakir Hussain, Harshit Kumar, Vikram Ramesh, Samir Das, Sandeep Ghatak, Shubham Loat, Martina Pukhrambam, Vijay Kumar Vidyarthi, Mihir Sarkar, Girish Patil Shivanagowda

**Affiliations:** 1 School of Engineering and Technology, Nagaland University, Kohima 797004, Nagaland, India; 2 Division of Animal and Fisheries Sciences, ICAR Research Complex for NEH Region, Umiam 793103, Meghalaya, India; 3 Department of Veterinary Public Health and Epidemiology, Karnataka Veterinary, Animal and Fisheries Sciences University, Vidyanagar, Hassan 573202, Karnataka, India; 4 ICAR-National Research Centre on Mithun, Medziphema 797106, Nagaland, India; 5 ICAR-National Research Centre on Yak, Dirang 790101, Arunachal Pradesh, India; 6 School of Agricultural Sciences, Nagaland University, Medziphema 797106, Nagaland, India

**Keywords:** colorimetric detection, isothermal, meat adulteration, mitochondrial *D-loop*, on-site diagnosis, yak meat

## Abstract

The growing demand for robust food authentication methods has driven the establishment of fast, sensitive, and field-based detection systems for identifying meat species. This study presents a colorimetric-based PSR approach for identifying yak (*Bos grunniens*) meat within fresh, thermally processed, and blended meat samples. Targeting the mitochondrial *D-loop* locus, the assay incorporates a simple alkaline lysis (AL) procedure for efficient DNA extraction, eliminating the requirement for specialized instrumentation. The PSR assay demonstrated high specificity, showing no evidence of cross-reactivity with closely associated food animals such as buffalo, cattle, goat, sheep, mithun, and pig. Sensitivity assessment revealed the assay’s capability to detect 1 pg of yak DNA, with reliable performance in samples exposed to thermal conditions up to 121 °C. Additionally, the technique detected yak meat down to a concentration of 0.1% in binary beef mixtures. This method provides a significant improvement in sensitivity over end-point PCR and is particularly well-suited for field applications due to its practical simplicity, affordability, as well as no reliance on sophisticated instrument. This is, to the best of our understanding, the first reported PSR-based approach developed for the identification of yak meat, offering a robust tool for food origin verification, regulatory enforcement, and product integrity monitoring.

## 1. Introduction

In recent years, the validation of meat product authenticity has gained increasing attention due to concerns related to food safety, economic fraud, and the protection of consumer rights. Species misidentification, whether through intentional substitution or accidental mislabeling, poses significant challenges to both meat industry and consumers [1,2]. For individuals with specific dietary restrictions, ethical concerns, or religious obligations, accurate species identification is particularly critical. Consequently, robust methods for meat species authentication are essential to ensure product integrity and uphold public trust [3,4,5].

Yaks (*Bos grunniens*) play a vital role in sustaining the livelihoods of pastoral populations inhabiting high mountainous zones of the Himalayas and the Tibetan plateau, representing an economically and culturally vital livestock species [6]. They contribute to ecological stability, food security, socio-economic development, and the preservation of highland cultural identity [7]. Yak meat is considered nutritionally superior, characterized by low fat content, high protein density, and an enriched profile of essential amino acids (EAAs) and health-promoting n-3 polyunsaturated fatty acids (PUFAs) [8]. Moreover, consumers often perceive yak meat as free from veterinary drug residues or chemical contamination. Despite its higher cost, yak meat and its derivatives remain popular food choices, though their availability is seasonal and yields are comparatively lower than beef from cattle (*Bos taurus*) [9]. Food fraud incidents, such as the replacement of regular beef over yak meat, are commercially motivated and mislead consumers, undermining the genuine yak meat market [10]. In order to uphold consumer confidence and maintain the integrity of yak meat, there is a critical need for a fast, reliable, and accurate technique to distinguish it from other domestic bovine species. Such tools are important for preventing contamination and ensuring the quality and traceability of meat products.

Over the past several decades, a wide array of species identification approaches has been developed, primarily focusing on protein- and nucleic acid-based markers. Protein-centric techniques such as ELISA [11] and proteomic profiling [12] have served as dependable tools and have been commercialized into detection kits for species adulteration. However, their effectiveness is limited in heat-treated or processed products due to protein denaturation. In contrast, DNA-based methods including PCR [2], real-time PCR [13], DNA barcoding [14], and digital PCR [15] have gained wider acceptance due to the inherent universality, high specificity, and superior stability of genetic material. These approaches are presently considered the reference method for accurately determining the species origin of meat products [16]. Despite their advantages, these methods often involve electrophoretic procedures, require specialized equipment, and demand high technical expertise, making them time-consuming, labor-intensive, and generally limited to well-equipped laboratories [17]. Moreover, their dependence on specialized variable-temperature equipment further constrains their use in field or low resource settings. On the contrary, isothermal nucleic acid amplification offers a significant advantage, enabling efficient DNA amplification at a constant temperature in a simple setup without the need for sophisticated instrumentation or extensive technical training.

**Figure 1:**
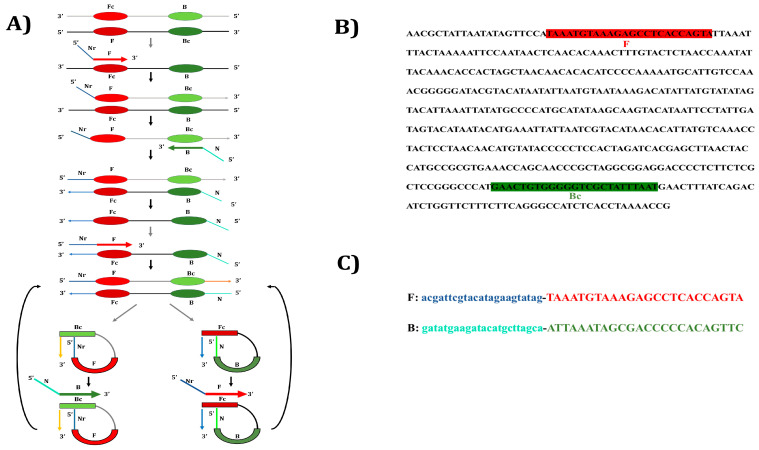
Diagrammatic representation. (**A**) PSR principle: The reaction is initiated when the target template is primed by forward and reverse primers that each contain an added 5′ tail-one in the sense orientation and the other in the reverse orientation. The resulting double-stranded product can subsequently fold into loop-like structures, which facilitate efficient primer annealing and drive exponential amplification. (**B**) Localization of primers on the *D-loop* segment of *Bos grunniens* (Gen Bank Accession number AY521161.1). (**C**) PSR oligonucleotides.

Among existing isothermal amplification platforms, the polymerase spiral reaction (PSR) [18] has emerged as a robust and efficient method for nucleic acid amplification (Figure 1). The mechanism of PSR, reinterpreted by Glökler and co-workers [18], suggests that a distinctive two-loop structure forms within the double-stranded intermediate, enabling effective primer binding and driving exponential amplification through continuous loop unwinding. This interpretation aligns with established biochemical principles and provides a rationale for the observation that PSR can reliably yield accurately sequenced and functional amplification products, despite the mechanism not being fully elucidated. Relying solely on *Bst* DNA polymerase, PSR operates under constant temperature conditions using simple equipment such as water bath or dry heat block. Compared with other isothermal approaches such as LAMP [19], RCA [20] and HDA [21], PSR offers simpler primer design, improved reaction stability, and high sensitivity and specificity. Its amplification products can be visualized through gel electrophoresis, fluorescence measurement, or colorimetric/fluorescent dyes including SYBR Green I, HNB, malachite green, calcein and phenol red [22]. PSR has been successfully applied for identifying diverse microbial pathogens [8,23,24] and for meat species authentication [25,26,27,28]. However, research on yak meat authentication remains limited, with most studies still relying on conventional molecular techniques such as PCR [29], DNA barcoding [30], real-time PCR [16] and PCR-RFLP [31], while only a few have evaluated isothermal methods like RPA [32] and LAMP [33]. 

While isothermal amplification techniques such as RPA and LAMP are highly sensitive and powerful, they often suffer from limitations including assay complexity, high cost, sensitivity to reaction conditions, intricate primer design, and the need for additional denaturation steps [34]. In contrast, PSR provides a simpler, more robust, and field-deployable alternative for meat species identification. Notably, no published studies to date have reported the application of PSR for yak meat authentication. In this study, we established and validated a PSR assay for yak meat identification by integrating alkaline lysis-based DNA isolation, amplification via PSR, fluorometric analysis, and dye-based detection in raw, thermally processed and meat mixture samples.

## 2. Materials and Methods

### 2.1. Collection of Meat/Blood Samples

Whole blood was drawn from the jugular vein of six individual yaks sourced from ICAR-National Research Centre on Yak, Arunachal Pradesh, India. One meat tissue sample of yak was collected from verified local slaughterhouse in Arunachal Pradesh, India. Four sheep whole blood samples were collected from the jugular vein at the College of Veterinary Sciences and Animal Husbandry, Nagaland, India. One meat tissue sample each from cattle, buffalo, goat, and pig were procured from authenticated municipal butcher shops in Dimapur District, Nagaland, while tissue specimen from six individual Mithuns were procured from the certified local municipal slaughter facility in Dimapur. All samples were aseptically collected, properly labelled with date and origin. The samples were collected in pre-sterilized containers to prevent any external contamination and transferred while sustaining controlled cooling to ICAR-National Research Centre on Mithun, Nagaland, India where they were stored at −20 °C until further evaluation.

### 2.2. Extraction of Total DNA

The total DNA content was isolated by applying the alkaline lysis (AL) protocol [35]. Concisely, 500 mg of fresh meat was taken and homogenized with 4 mL of 0.2 mol/L NaOH solution (Merck, Rahway, NJ, USA) in an autoclaved pestle and mortar. Subsequently, 10 µL of the resulting extract was mixed with 40 μL of 0.2 mol/L NaOH and heated to 75 °C using a dry bath (Labnet International, Inc., Edison, NJ, USA) for a duration of 20 min. The thermal lysis was followed by neutralization of the reaction mixture by adding 360 μL of 0.04 mol/L Tris-HCl (Promega, Madison, WI, USA) buffer (pH 7.75), in accordance with the established procedure. Following extraction, the DNA was stored at −20 °C until further analysis. Prior to its use as a PSR template, its purity determined by the OD_260/280_ absorbance ratio, and its concentration were quantified using a NanoDrop 2000 spectrophotometer (Thermo Fisher Scientific, Waltham, MA, USA).

### 2.3. Designing of Nucleotide Primers for Yak PSR

A set of PSR primers was constructed targeting the yak mitochondrial *D-loop* region by appending stuffer sequences Nr and N [18] to the 5′ ends. The yak specific forward (F) and reverse (R) primers reported previously were utilized [2] and the stuffer sequences were annexed to these yak specific primers. In these primers, the uppercase sequences at the 3′ ends of both forward and reverse primers correspond to the target *D-loop* region of the yak mitochondrial genome, while the lowercase 5′ regions, derived from a plant gene, are reverse to one another. Oligonucleotides used in this study were custom-synthesized by Barcode Biosciences (Bangalore, Karnataka, India). The details of the primer used for PSR are provided in Table 1.

### 2.4. Yak PSR Assay Standardization

The effects of critical reaction components and procedural conditions on PSR assay efficiency and reliability were systematically assessed. Standardization procedures were carried out for primers (8–20 µM), MgSO_4_ (2–10 mM) (NEB, Ipswich, MA, USA), betaine (0–1.0 M) (Sigma-Aldrich, Burlington, MA, USA), dNTPs (0.8–1.6 mM) (ThermoFisher, Waltham, MA, USA), *Bst* 3.0 DNA Polymerase (6–14 U) (NEB, Ipswich, MA, USA), reaction temperature (60–65 °C) and incubation time (15–90 min). The PSR amplification products were evaluated visually by monitoring changes in color and fluorescence intensity. To the completed reaction, 1 μL of SYBR Green I nucleic acid stain (1:10 dilution of 10,000× in DMSO; Invitrogen, ThermoFisher Scientific, Waltham, MA, USA) was incorporated to the reaction mixture. A green fluorescence indicated a positive PSR, whereas an orange hue denoted a negative outcome.

### 2.5. Detection and Confirmation of PSR Results Through Agarose Gel Separation and Fluorometric Analysis

Validation of PSR amplification was performed through 1.5% agarose gel electrophoresis, with visualization using a gel imaging system (Vilber Lourmat, Vilber, Collégien, France). Following the addition of SYBR Green I to the final reaction mixture, fluorescence intensity was quantified using a CFX Opus 96 Real-Time PCR instrument (Bio-Rad, Hercules, CA, USA) and fluorescent intensity values were noted in terms of Relative Fluorescence Units (RFU).

### 2.6. Detection Limit and Selectivity Evaluation for PSR

To determine assay sensitivity, yak meat genomic material was processed through a series of ten-fold dilutions with nuclease-free water, covering a concentration range from 100 ng/μL to 100 ag/μL. Amplification was performed under optimized PSR conditions for each dilution.

To assess specificity, the PSR assay was challenged with genomic DNA from cross species, including mithun, cattle, buffalo, pig, goat, and sheep. The absence of amplification in other closely related species confirmed the assay’s strong specificity and its targeted application for yak meat identification.

### 2.7. Assessment of PSR Assay Reliability in Heat-Processed and Mixed Meat Mixtures

To assess the influence of heat treatment on PSR assay efficiency, yak meat samples were exposed to standardized thermal processing conditions. Precisely weighed portions of raw meat (500 mg) were incubated at 60 °C, 80 °C, 100 °C and 121 °C for half an hour in a dry heat block. These temperature conditions reflect typical culinary practices, with 121 °C representing the maximum temperature normally encountered during routine cooking. Following heat treatment, total genomic DNA was isolated from each sample adopting the AL method, after which the PSR assay was conducted to evaluate its efficacy for identifying target DNA under varying levels of thermal exposure.

To investigate the capability of the PSR assay to detect meat mixtures, binary meat admixtures were prepared using raw yak and cattle meat. Each admixture weighed a total of 500 mg, with yak-to-cattle meat ratios (*w*/*w*) of 20:80, 10:90, 5:95, 1:99, 0.5:99.5, and 0.1:99.9. The samples were thoroughly homogenized to ensure uniform distribution of yak tissue within the cattle matrix. Total DNA was isolated from each admixture utilizing the AL method described previously. The extracted DNA was then subjected to PSR analysis to evaluate the assay’s sensitivity and establish the minimum detectable concentration of yak genomic content in the mixed meat samples.

### 2.8. Statistical Evaluation

To establish the cut-off values for the yak-specific PSR assay, mean fluorometric readings obtained from PSR products of non-target food animals (mithun, buffalo, cattle, pig, sheep, and goat) were utilized [36]. Each factor was evaluated derived from the average of three experimental runs, and the resulting measurements were processed using Microsoft Excel (Microsoft Corporation, Redmond, DC, USA).

## 3. Results and Discussion

### 3.1. DNA Quantity and Purity

Nucleic acid isolated from both fresh and heat processed yak meat employing the AL method exhibited quantity and purity levels appropriate for PSR amplification. Mean DNA yields from raw meat and from samples heated to 60 °C, 80 °C, and 100 °C, and 121 °C were 138.1, 39.7, 44.1, 41.2, and 45.1 ng/μL, respectively, with corresponding OD_260/280_ ratios ranging from 1.34 to 1.72. These values denote sufficient DNA integrity for downstream applications, although variability in yield and quality were noted with increasing thermal treatment. Comparable observations have been documented in studies involving raw and cooked pork [37], carabeef [38], and mutton [39].

Conventional DNA purification procedures, such as the phenol: chloroform technique, are well-regarded for their high performance and affordability; however, their reliance on hazardous organic solvents and labour-intensive protocols poses significant safety and practical challenges [35]. In comparison, ready-to-use nucleic acid isolation systems deliver enhanced protection and yield which tends to be not only economically unfeasible but also involve multiple centrifugation steps, thus limiting their suitability for rapid or field-based applications. In this study, the AL method served as a feasible and accessible choice, offering a simplified, single-tube protocol that depends on standard laboratory chemicals. This single-reaction method minimizes contaminant carryover risks while preventing the need for centrifugation, making it ideal for quick, field-based diagnostic purposes [40]. Its demonstrated capability to isolate DNA from fresh as well as heat-treated meat underscores its potential use in verifying food authenticity and in forensic analysis.

### 3.2. Standardization of PSR Assay Parameters

Following a comprehensive reagent optimization, the finalized 25 µL polymerase spiral reaction (PSR) test mixture was prepared with the following composition: 2.5 µL of 10X Isothermal amplification buffer, 10 µM of both forward and backward primers, 4 mM magnesium sulphate (MgSO_4_), 1.4 mM deoxynucleotide triphosphates (dNTPs), 0.8 M betaine, 8 units of *Bst* 3.0 DNA polymerase, and 3 µL of DNA template, with the remaining volume adjusted to 25 µL using nuclease-free water. The standardized amplification was conducted at 64 °C for 75 min.

#### 3.2.1. Primer Dosage

**Figure 2:**
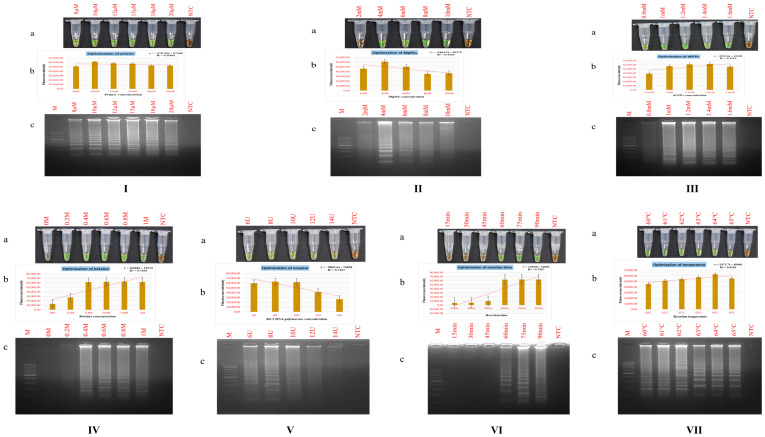
Optimization of PSR reagents. (**I**) Primer. (**II**) MgSO_4_. (**III**) dNTP. (**IV**) Betaine. (**V**) *Bst* 3.0 DNA polymerase. (**VI**) Time. (**VII**) Temperature. Lane NTC: non-template control. Lane M: 100 bp DNA marker. (**a**) Colorimetric detection. (**b**) Fluorescence measurements. (**c**) Gel electrophoresis patterns of PSR products.

To identify the most effective primer concentration for the PSR technique, primers were evaluated across a concentration range of 8 µM to 20 µM. A transition to green colour following the addition of SYBR Green I fluorophore indicated detective amplification in positive samples (Figure 2I(a)). Within the examined concentration series, 10 µM yielded the maximum fluorescence signal (Figure 2I(b)), indicating superior amplification efficiency. Agarose gel analysis showed a uniform ladder-like banding patterns across all concentrations (Figure 2I(c)), confirming reliable product generation. Nonetheless, fluorometric measurement identified 10 µM as the most effective concentration under the assay parameters. The results are consistent with previous observations, highlighting that maintaining an optimal primer concentration is critical for PSR efficiency as it ensures accurate target hybridization, minimizes nonspecific amplification, and improves both reaction performance and yield [41].

#### 3.2.2. Magnesium Sulphate (MgSO_4_) Dosage

Various concentrations of magnesium sulphate (2–10 mM) were assessed to identify the optimal condition for the PSR assay. A noticeable green hue change, indicative of successful amplification, was noted at concentrations ranging from 4 to 10 mM, while no such colour shift was evident at 2 mM (Figure 2II(a)). Fluorometric quantification revealed the highest signal at 4 mM, with a gradual decline at 6, 8, and 10 mM, establishing 4 mM as the ideal concentration (Figure 2II(b)). Electrophoretic gel analysis confirmed ladder-like amplification bands under all tested concentrations except 2 mM, with a noticeable reduction in amplification efficiency beyond 6 mM (Figure 2II(c)). Magnesium sulphate is essential for regulating the activity of *Bst* DNA polymerase. Previous reports have demonstrated that lower MgSO_4_ amounts, when combined with elevated levels of *Bst* polymerase, can promote DNA synthesis performance and improve detectability threshold in PSR and various other isothermal amplification systems [42,43]. Nevertheless, excessive MgSO_4_ may prevent enzymatic function of the polymerase and disrupt the DNA structure, resulting in reduced amplification efficiency. These findings are consistent with earlier studies employing 4 mM MgSO_4_ in PSR amplification of diverse genes [44,45]. Nevertheless, higher levels of MgSO_4_, such as 16 mM, have also been reported under specific assay settings [46].

#### 3.2.3. Deoxynucleotide Triphosphates (dNTPs) Dosage

The impact of varying dNTP concentrations (0.8–1.6 mM) on PSR amplification efficiency was determined to optimize assay conditions. Observable colour transition was noted across all concentrations (Figure 2III(a)), with the highest fluorescence intensity noted at 1.4 mM, indicating it as the most effective concentration under the given conditions (Figure 2III(b)). Electrophoretic analysis generated prominent laddering profiles between 1.0 and 1.6 mM, whereas a comparatively weak signal was detected at 0.8 mM (Figure 2III(c)). These observations corroborate previous findings that employed higher dNTP concentrations for efficient amplification [22,47]. In contrast, other studies have successfully used lower concentrations, such as 1.2 mM [27,28], highlighting that optimal dNTP levels are dependent on the nature of the target sequence and specific reaction conditions [48]. Notably, the required dNTP concentration in PSR assay is governed by variables including primer length, magnesium ion levels, and operating temperature [49].

#### 3.2.4. Betaine Dosage

PSR amplification was evaluated across betaine levels ranging from 0 to 1 M. Upon the addition of SYBR Green I, samples containing 0.1–1.0 M betaine exhibited a vivid green coloration, whereas the 0 M dosage showed no visible colour change (Figure 2IV(a)). Quantitative fluorometric analysis indicated the highest signal at 0.8 M betaine (Figure 2IV(b)). Consistently, agarose gel separation revealed a clear laddering signal between 0.4 and 1 M, with no detectable amplification in the absence of betaine (Figure 2IV(c)). Although intensive bands appeared at both 0.6 and 0.8 M, the latter was established as the ideal condition based on superior fluorescence measurements. Betaine, a zwitterionic osmolyte at near-neutral pH, facilitates uniform DNA melting by stabilizing strand denaturation and reducing AT/GC melting temperature differentials [50]. Within the PSR operational temperature range of 61–65 °C, it facilitates efficient helix unwinding, minimizes secondary-structure formation, and enhances primer–template hybridization selectivity [51]. The standardized 0.8 M concentration established here aligns to those reported in earlier PSR optimization conditions targeting diverse genes, further corroborating its broad applicability in isothermal amplification assays [24,26].

#### 3.2.5. *Bst* DNA Polymerase Dosage

To determine the best effective concentration of *Bst* DNA polymerase for the yak PSR assay, enzyme levels of 6, 8, 10, 12, and 14 U were evaluated. Appearance of fluorescent green coloration, indicative of positive amplification, was visualized at concentrations ranging from 6 to 12 U, while only a faint color shift was noted at 14 U (Figure 2V(a)). Fluorescence analysis revealed the maximum fluorescence signal at 8 U, determining this concentration to be the most effective for target amplification (Figure 2V(b)). Consistently, gel electrophoresis showed a distinct ladder-like banding pattern at 8 U, with diminishing band intensities at 10 and 12 U, and no visible amplification at 14 U (Figure 2V(c)). Considering these results, 8 U was chosen as the most suitable concentration of enzyme for the assay. These results correspond with the recognized role of magnesium ions (Mg^2+^) in regulating *Bst* DNA polymerase function, as Mg^2+^ facilitates the effective binding of nucleoside triphosphates (NTPs) during the replication of DNA [52]. The 8 U enzyme level likely ensured an optimal Mg^2+^ to enzyme ratio, promoting efficient amplification while minimizing background noise. This balance enhanced the detection limit and assay exclusivity of the PSR technique, underscoring the robustness of the optimized reaction conditions.

#### 3.2.6. Reaction Time and Temperature

To optimize the reaction duration for the PSR assay, incubation times ranging from 15 to 90 min were evaluated. Visual analysis employing SYBR Green I dye did not reveal any color change at 15, 30, and 45 min. Conversely, a positive green color shift, reflective of successful amplification, was observed at 60, 75, and 90 min (Figure 2VI(a)). Maximum fluorescence intensity was recorded at 75 min (Figure 2VI(b)), which was further corroborated by a prominent ladder-like bands on agarose gel electrophoresis (Figure 2VI(c)). Based on these results, 75 min was set as the ideal incubation period for the yak PSR. This finding is consistent with previous investigations reporting effective amplification at elevated thermal conditions (63–66 °C) combined with extended incubation periods for different targets [23,53,54]. Conversely, some studies have demonstrated efficient amplification within a shorter time frame [55,56]. These variations are in accordance with the heat-resistant property of *Bst* DNA polymerase allowing it to exhibit maximal function in the 60–65 °C scale [57]. The parameters optimized in the present study ensure efficient amplification while maintaining assay time, resulting in consistent and reliable outcomes.

To optimize the PSR method, a series of temperature settings ranging from 60 to 65 °C was evaluated. Detectable colorimetric changes were observed across this temperature range upon SYBR green I dye addition (Figure 2VII(a)). Fluorometric analysis identified 64 °C as the temperature yielding the highest signal intensity (Figure 2VII(b)). Consistent with these findings, agarose gel electrophoresis demonstrated comparable amplification profiles across the temperature gradient (Figure 2VII(c)), confirming 64 °C as the most effective incubation temperature for PSR amplification.

### 3.3. Assessment of PSR Assay Sensitivity and Selectivity Parameters

**Figure 3:**
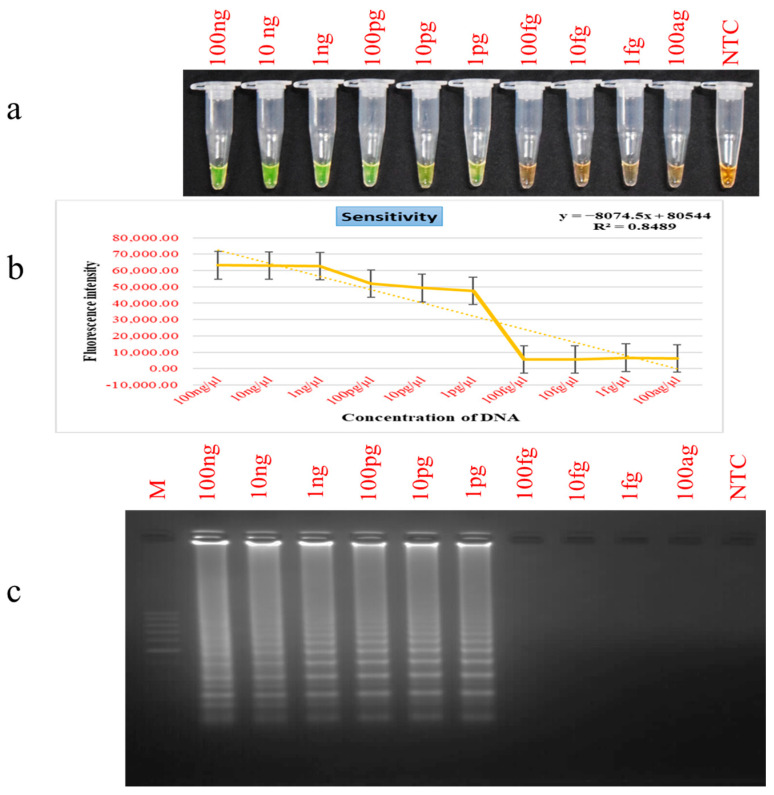
Analytical sensitivity. Lane NTC: Non-template control. Lane M: 100 bp DNA marker. (**a**) Colorimetric detection. (**b**) Fluorescence measurements. (**c**) Gel electrophoresis patterns of PSR products.

The limit of detection (LOD) was determined to evaluate the sensitivity of the PSR assay. A series of ten-fold serial dilutions of yak genomic DNA, ranging from 100 ng/µL to 100 ag/µL, were prepared and subjected to amplification. Visual colour analysis (Figure 3a) and gel electrophoretic analysis (Figure 3c) displayed a gradual decline in amplification intensity with decreasing DNA concentrations, showing no amplification detectable lower than 1 pg. Therefore, the LOD of the PSR assay was established at 1 pg. In contrast, the endpoint PCR exhibited a lesser sensitivity, identifying yak DNA only down to 100 pg [2]. This demonstrates the superior sensitivity of the method, which is approximately 100 times more sensitive than conventional PCR. The LOD obtained in this study aligns with that reported in the LAMP-HNB assay for yak meat identification [33]. The high sensitivity and rapid performance of PSR assays are mainly attributed to the use of specifically designed primers, which frequently demonstrate superior efficiency compared to other amplification methods [47]. Furthermore, the observed reduction in amplification intensity with decreasing template concentrations further supports quantitative, concentration-dependent response. While other studies have reported lower detection limits, for example, 0.1 pg for LAMP [58] and 0.76 pg for PSR assays [26], for detecting other species, the current PSR assay demonstrates high sensitivity for yak DNA detection, making it well-suited for practical applications.

**Figure 4:**
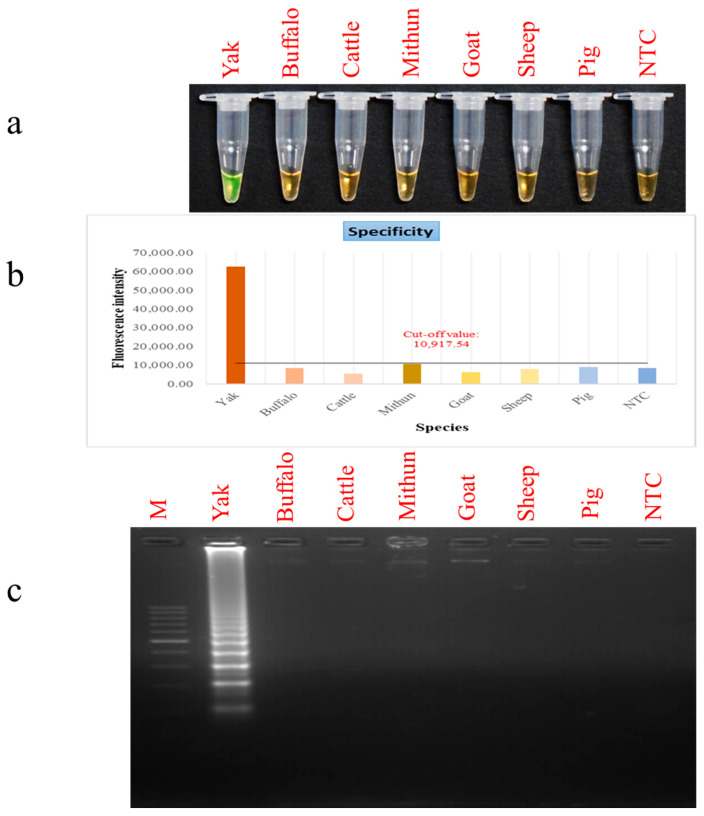
PSR assay specificity. Lane NTC: non-template control. Lane M: 100 bp DNA marker. (**a**) Colorimetric detection. (**b**) Fluorescence measurements. (**c**) Gel electrophoresis patterns of PSR products.

The newly developed PSR assay, targeting the *D-loop* region of yak mtDNA, demonstrated high analytical specificity, with no cross-reaction observed in closely associated livestock animals such as buffalo, cattle, mithun, goat, sheep, and pig. This marked specificity is critical for applications in food verification, forensic examinations, and genetic conservation studies. No evidence of non-target amplification confirms the robustness of the oligonucleotide design in discriminating yak DNA from that of genetically related species. Amplification in the species of interest was indicated by development of green fluorescence, whereas non-target species yielded an orange colour, denoting negative results (Figure 4a). These visual findings were substantiated by fluorometric analysis, which established a fluorescence cut-off value of 10,917.54. This value was derived from the mean fluorescence of non-target species (μ = 7858.56) and the standard deviation (σ = 1859.56) using the Z-score at the 95th percentile [36] (Figure 4b). Validation by agarose gel electrophoresis further confirmed the assay’s specificity (Figure 4c).

Fluorometric readings for yak meat were consistently above the threshold, affirming the reproducibility and specificity of the assay. This robustness illustrates its real-world applicability for food origin authentication, particularly in regulatory compliance implementation and routine quality control. Additionally, the high-intensity fluorescence signals indicate the assay’s capability to detect low concentrations of yak DNA, further underscoring its sensitivity. These results are consistent with previous studies advocating for isothermal amplification techniques as effective tools for verifying species origin owing to their speed, sensitivity, and low instrumentation demand.

### 3.4. Evaluation of PSR Performance in Thermally Processed Meat Matrices

**Figure 5:**
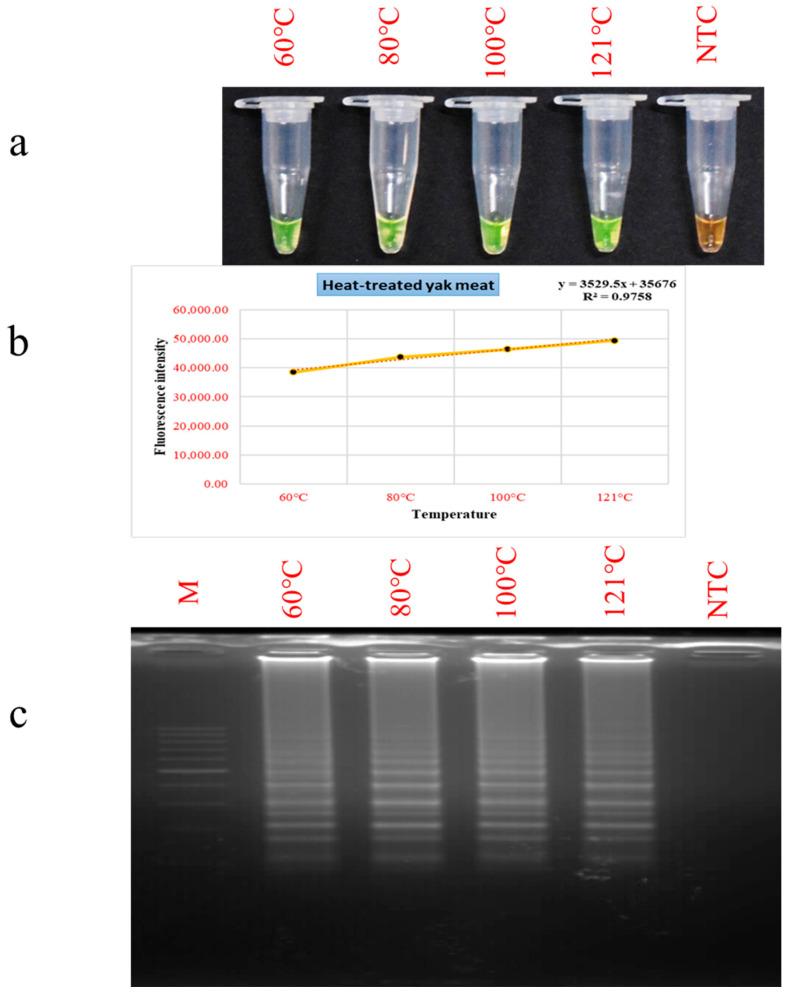
Detection of heat-processed yak meat samples using the PSR assay. Lane NTC: non-template control. Lane M: 100 bp DNA marker. (**a**) Colorimetric detection. (**b**) Fluorescence measurements. (**c**) Gel electrophoresis patterns of PSR products.

The PSR assay’s tolerance to thermal processing was examined using raw yak meat heated to 60 °C, 80 °C, 100 °C, and 121 °C for half an hour. Distinct green colorimetric signals were observed in all treated samples (Figure 5a), indicating successful amplification and confirming the assay’s capability to identify yak DNA in cooked meat. Fluorometric analysis and gel electrophoresis further supported these observations, showing increased fluorescence intensity and amplification signals with higher processing temperatures (Figure 5b,c). This increase is likely attributable to thermal cellular membrane damage, which promotes enhanced DNA recovery and accessibility [59,60]. These results are consistent with earlier reports of LAMP-based detection in cooked buffalo meat and detection of chevon using the PSR assay in thermally treated samples [25,38]. The ability to detect yak DNA under such rigorous conditions highlights the assay’s relevance for food authenticity testing, particularly in processed meat products where DNA degradation can pose significant analytical challenges.

The PSR assay reliably amplified yak DNA from meat samples subjected to thermal processing at 121 °C for 30 min, as well as from samples heated to 60 °C, 80 °C, and 100 °C for the same time period. The assay’s capability to retain reliable amplification signals across a broad range of thermal conditions underscores its stability, indicating its suitability for application in both raw and processed meat analysis. This adaptability is crucial for the practical adoption of the PSR method in routine meat processing sector testing, regulatory oversight, and quality assurance.

### 3.5. Evaluation of PSR Assay for Meat Admixture Detection

**Figure 6:**
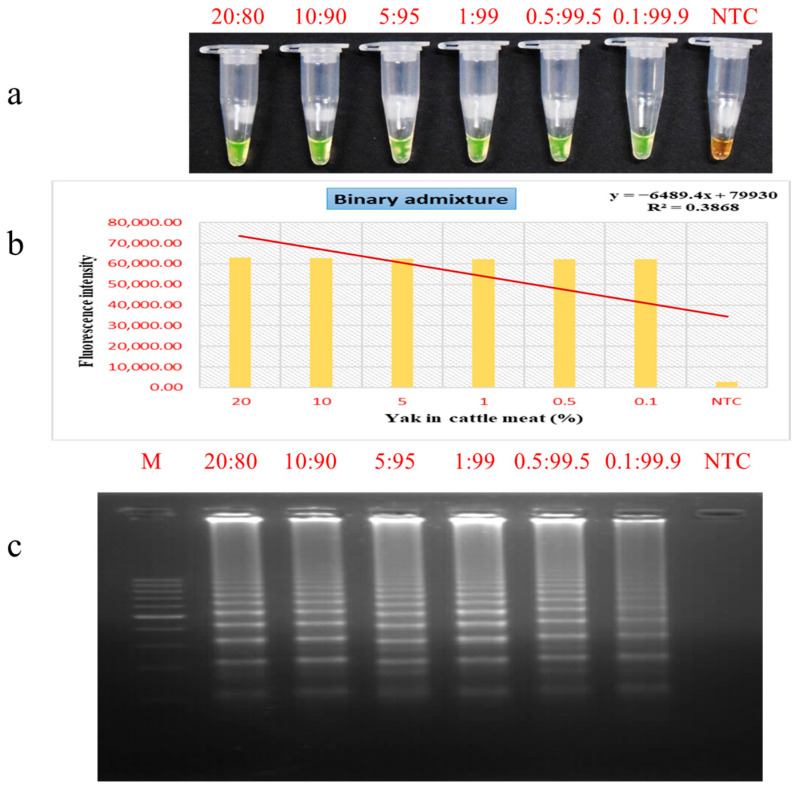
Detection of yak DNA in binary yak-beef mixtures using the PSR assay. Lane NTC: non-template control, Lane M: 100 bp DNA marker. (**a**) Colorimetric detection. (**b**) Fluorescence measurements. (**c**) Gel electrophoresis patterns of PSR products.

The PSR assay demonstrated strong potential for identifying yak meat in binary mixtures with cattle, as indicated by varying intensities of green coloration across different blend ratios (Figure 6a). Sensitivity assessment through agarose gel electrophoresis confirmed the assay’s ability to identify yak DNA down to a concentration of 0.1% in beef (Figure 6c), highlighting its applicability for identifying both deliberate and unintentional adulteration in meat products. Comparable sensitivity has been reported in earlier studies using LAMP and RPA methods, where pork was detected in beef at 0.1% [37] and duck meat in mixed powders at similar levels [61]. The effectiveness of DNA-based meat identification techniques is influenced by multiple factors, including primer design, species-specific changes in DNA content, and the efficiency of DNA isolation protocols. The latter, in particular, can be challenging due to differences in meat types along with elevated lipid levels in certain meats, which can hinder DNA isolation efficiency and give rise to inaccurate quantification [62,63]. In comparison with a recent report [40], which demonstrated that a duplex RPA-CRISPR/Cas12a assay could detect yak–cattle meat mixtures at levels as low as 1%, the PSR assay developed in this study exhibited superior sensitivity. In a related study, the RPA-LF assay was shown to detect pig-derived tissues at a threshold level of 1% [64], thereby highlighting the superior sensitivity of the PSR assay in detecting low-level adulteration. Advanced techniques such as biosensor chips based on optical thin films exhibited exceptional sensitivity down to 0.001% for deer and beef in pork powder [65], and LAMP assay had LODs from 0.01% to 0.0001% levels [60], but these methods often require expensive instrumentation, controlled laboratory environments, and are prone to contamination, limiting their practicality for on-site testing. On the other hand, the PSR assay combines high sensitivity with simplicity and portability, offering a cost-effective and field-friendly alternative. Its minimal equipment requirements and rapid turnaround time make it especially useful for real-time food authentication and enforcement activities. These attributes position the PSR assay as a powerful tool for combating both deliberate and accidental meat adulteration, helping to uphold food integrity, protect consumer trust, and support regulatory compliance.

## 4. Conclusions

The present work introduces a rapid, in-field isothermal principle-based PSR visualization assay for the identification of yak meat, aligning with the WHO’s “ASSURED” criteria (affordable, sensitive, specific, user-friendly, robust, rapid, equipment-free, and deliverable) for diagnostic tools. When paired with a simple and efficient alkaline lysis (AL) sample preparation method, the assay enables rapid and accurate identification of yak meat. Comprehensive performance evaluations demonstrated the assay’s high specificity for yak, exceptional sensitivity (1 pg DNA), and robustness, with the ability to detect yak DNA in thermally treated samples (up to 121 °C) and yak admixtures as low as 0.1% in cattle meat. To the best of our understanding, this is the first reported application of a PSR assay for yak meat detection. Furthermore, the methodology holds promise for broader applications, including the detection of yak ingredient in food products. The proposed assay is a simple, reliable, fast, and economical solution that does not require sophisticated instrumentation, rendering it highly suitable for field-based meat authenticity investigation in food safety and regulatory enforcement settings.

## Figures and Tables

**Table 1:** Primer sequence used for yak polymerase spiral reaction (PSR).

Region	Technique	Oligonucleotide Sequence (5′-3′)	Amplicon Size	Reference
***D-loop***	PCR-F	TAAATGTAAAGAGCCTCACCAGTA	422 bp	[2]
	PCR-R	ATTAAATAGCGACCCCCACAGTTC		
	PSR-F	acgattcgtacatagaagtatagTAAATGTAAAGAGCCTCACCAGTA	variable	
	PSR-R	gatatgaagatacatgcttagcaATTAAATAGCGACCCCCACAGTTC		

The lowercase sequences are external sequences.

## Data Availability

The original contributions presented in this study are included in the article. Further inquiries can be directed to the corresponding authors.
